# Identification of Phosphodiesterase-7A (PDE7A) as a Novel Target for Reducing Ethanol Consumption in Mice

**DOI:** 10.1093/ijnp/pyae032

**Published:** 2024-08-05

**Authors:** Ran Wei, Fangjiao Zong, Jiahao Dong, Wei Zhao, Fangfang Zhang, Wei Wang, Shuang Zhao, Ziqi Wang, Fang Zhang, Han-Ting Zhang

**Affiliations:** Department of Pharmacology, Qingdao University School of Pharmacy, Qingdao, China; Weifang Chinese Medical Hospital, Shandong Second Medical University, Weifang, China; Department of Pharmacology, Qingdao University School of Pharmacy, Qingdao, China; Department of Pharmacology, Qingdao University School of Pharmacy, Qingdao, China; Weifang People’s Hospital, Shandong Second Medical University, Weifang, China; Department of Pharmacology, Qingdao University School of Pharmacy, Qingdao, China; Institude of Pharmacology, Shandong First Medical University and Shandong Academy of Medical Sciences, Tai’an, China; Institude of Pharmacology, Shandong First Medical University and Shandong Academy of Medical Sciences, Tai’an, China; Department of Pharmacology, Qingdao University School of Pharmacy, Qingdao, China; Department of Pharmacology, Qingdao University School of Pharmacy, Qingdao, China; Department of Pharmacology, Qingdao University School of Pharmacy, Qingdao, China; Department of Pharmacology, Qingdao University School of Pharmacy, Qingdao, China

**Keywords:** Alcoholism, cAMP-PKA/Epac2 pathway, EtOH responsiveness, PDE7A, sex difference

## Abstract

**Background:**

Ethanol elicits a rapid stimulatory effect and a subsequent, prolonged sedative response, which are potential predictors of EtOH consumption by decreasing adenosine signaling; this phenomenon also reflects the obvious sex difference. cAMP (cyclic Adenosine Monophosphate)-PKA (Protein Kinase A) signaling pathway modulation can influence the stimulatory and sedative effects induced by EtOH in mice. This study’s objective is to clarify the role of phosphodiesterase (PDE) in mediating the observed sex differences in EtOH responsiveness between male and female animals.

**Methods:**

EtOH was administered i.p. for 7 days to identify the changes in PDE isoforms in response to EtOH treatment. Additionally, EtOH consumption and preference of male and female C57BL/6J mice were assessed using the drinking-in-the-dark and 2-bottle choice tests. Further, pharmacological inhibition of PDE7A heterozygote knockout mice was performed to investigate its effects on EtOH-induced stimulation and sedation in both male and female mice. Finally, Western blotting analysis was performed to evaluate the alterations in cAMP-PKA/Epac2 pathways.

**Results:**

EtOH administration resulted in an immediate upregulation in PDE7A expression in female mice, indicating a strong association between PDE7A and EtOH stimulation. Through the pharmacological inhibition of PDE7A KD mice, we have demonstrated for the first time, to our knowledge, that PDE7A selectively attenuates EtOH responsiveness and consumption exclusively in female mice, whichmay be associated with the cAMP-PKA/Epac2 pathway and downstream phosphorylation of CREB and ERK1/2.

**Conclusions:**

Inhibition or knockdown of PDE7A attenuates EtOH responsivenessand consumption exclusively in female mice, which is associated with alterations in the cAMP-PKA/Epac2 signaling pathways, thereby highlighting its potential as a novel therapeutic target for alcohol use disorder.

**Significance Statement**

Alcohol dependence (alcoholism) is a psychiatric disease affecting hundreds of millions of people worldwide. And there are studies that the stimulating and sedative effects of acute EtOH consumption are significantly different between different genders. In the present study, EtOH administration resulted in an immediate upregulation in PDE7A expression in female mice, indicating a strong association between PDE7A and EtOH stimulation. We have demonstrated for the first time, to our knowledge, that PDE7A selectively attenuates EtOH responsiveness and consumption exclusively in female mice, which may be associated with cAMP-PKA/Epac2 pathway. Our study findings underscore the therapeutic potential of targeting PDE7A as a promising treatment modality for alcohol use disorder, aiming to facilitate translational research for gender-specific clinical interventions.

## INTRODUCTION

According to the statistics reported by the World Health Organization, approximately 2 billion individuals worldwide consume alcohol, and alcohol-related diseases contribute to approximately 6% of the total annual deaths globally (Fairbanks et al., 2020). Substantial inter-individual variability in terms of ethanol (EtOH) effects, such as stimulation (e.g., excitement) and sedation (e.g., sleepiness), has been reported (Ray et al., 2010). These factors hold promise as valuable indicators for investigating alcohol use disorder (AUD) (Ray et al., 2016) and assessing alcohol drinking–related risks (Newlin and Thomson, 1990).

Additionally, the drinking-in-the-dark (DID) paradigm has been employed in various studies to induce high levels of EtOH intake and blood ethanol concentration (BEC) in mice within a short duration, serving as a fundamental indicator of AUD (Thiele and Navarro, 2014; Bowen et al., 2022). Sex also plays a significant role in these aforementioned factors influencing EtOH intake; however, its impact remains controversial across different studies (Cunningham and Shields, 2018; Leyrer-Jackson et al., 2022). Notably, female mice exhibited significantly higher EtOH intake and preference compared with males (Fulenwider et al., 2019; Suárez et al., 2020).

The intracerebroventricularly administration of SP-cAMP before the hypnotic dose of EtOH reportedly resulted in the augmentation of PKA activity and prolongation of the loss of right reflex (LORR) duration induced by ethanol (Kumar et al., 2012). The enhanced stimulatory effects and reduced sedative effects of EtOH reportedly are associated with increased alcohol consumption (Castello et al., 2015, 2016). EtOH induces phosphorylation of the cAMP response element binding protein (CREB) by promoting PKA activation (Asyyed et al., 2006) and facilitating the nuclear translocation of intracellular Ca2+ in a cascade manner (Baliño et al., 2014). Extracellular signal-regulated protein kinases (ERKs) are also involved in the changes of protein molecules and synaptic plasticity in the dorsal striatum following chronic ethanol exposure (Cui et al., 2011). Overall, the cAMP signaling pathway exhibits significant relevance to EtOH consumption behavior and holds potential as a regulatory mechanism for AUD.

PDEs, a superfamily of enzymes, are classified into 11 families (PDE1–11) based on their substrate and inhibitor sensitivities, kinetic properties, allosteric regulation, and amino acid sequences (Beavo, 1995; Lugnier, 2006). PDE4 inhibitors, such as rolipram, also effectively reduce EtOH consumption in mice (Liu et al., 2017; Ozburn et al., 2020) and alcohol-preferring rats without altering the total amount of fluid intake (Hu et al., 2011). However, no study, to our knowledge, has explored the potential sex differences, which warrants further investigation and presents a promising avenue for future research. Furthermore, the emetic side effect of PDE4 inhibitors presents a significant obstacle (Heaslip and Evans, 1995) when considering PDE4 as a potential drug target for treating AUD. Contrarily, PDE7, the second family of PDE enzymes identified to specifically hydrolyze cAMP (Martinez and Gil, 2014), apparently is not involved in the emetic response (García et al., 2014). The inhibition of PDE7 has a protective effect on human dopaminergic nerve SH-SY5Y cells and increases neural stem cell proliferation and neuronal cell differentiation (Morales-Garcia et al., 2020).

PDE7 has been studied primarily for its role in neurodegenerative diseases such as Alzheimer disease (Safavi et al., 2013; Bartolome et al., 2018), but its role in mediating AUD has not been investigated. In the present study, we demonstrated that EtOH administration resulted in an immediate upregulation of PDE7A expression, indicating a strong association between PDE7A and EtOH stimulation. Additionally, using PDE7A KD mice or pharmacological inhibition of PDE7A selectively, we verified the participation of PDE7A in EtOH responsiveness and consumption exclusively in female mice, thereby highlighting the potential of PDE7A as a therapeutic target for AUD.

## MATERIALS AND METHODS

### Animals

The animals were housed in polypropylene plastic cages with a controlled temperature of 2°C ± 1°C and a 12:12-hour light cycle in single cages and provided ad libitum access to water and food pellets. All care and experimental studies of the animals were conducted following the Guide for the Care and Use of Laboratory Animals guidelines published by the US National Institutes of Health (publication no. 85-23, revised 1996).

We deleted an approximately 33-kb cluster of *PDE7A* genes on chromosome 3 in C57BL/6J mice by using the EGE (Biocytogen Extreme Genome Editing System) method with Cas9 proteins and 2 guide RNAs (gRNAs, Integrated DNA Technologies) targeting exon 2-13 coding region (Biocytogen Pharmaceuticals Co., Ltd, China). We verified the removal of the gene cluster in genetically modified mice using PCR. The primer sequences were WT-F (CAGTAATGTTCAGGTGGCGAAGGCT) and Mut-R (CAGCTAGGACAGTGGTGTTCCTTCC).

### Ethanol-Induced Locomotion

Mice were transferred to the experimental room and acclimated for 1 hour before they were placed in polycarbonate chambers (40 cm H × 40 cm W × 40 cm D) equipped with infrared photobeam detectors. The locomotor activity of male and female mice was counted at 5-minute intervals, with a saline injection administered 15 minutes before the baseline measurement and 40 minutes after (Phillips and Shen, 1996; Bocarsly, 2019). The mice received saline (i.p.) for 3 days to habituate to injection. Subsequently, mice were administered either saline or EtOH (2.5g/kg, i.p.) over 7 consecutive days. When testing the PDE7A inhibitor, mice received pretreatment with either saline or BRL50481 (5mg/kg, i.p.) 2 hours before receiving either EtOH or saline, then the trajectory of mice were recorded by Smart 3.0 software (Harvard Apparatus, Holliston, Massachusetts, USA) and analyzed automatically.

### Loss of Righting Reflex (LORR)

Mice were transferred to the experimental room and acclimated for 1 hour before they were injected with EtOH (3.5 g/kg, i.p.). They were placed supine on a smooth and flat board (Belknap and Akers, 1991). The time to lose the righting reflex, which was defined as the time from injection to when the mouse showed an inability to right itself within a 30 seconds time interval, was recorded. The mice were then left undisturbed until they began to regain the righting reflex. Once the mouse self-rights, it is again placed supine. When the mouse can self-right 3 times within 30 seconds, the time of righting is recorded. The LORR time was defined as the time between losing the reflex and regaining it. If the mice lost the righting reflex for >90 minutes, its data were excluded from the analysis.

### EtOH DID Test

The DID paradigm was performed starting at 3 hours after the onset of the dark cycle (lights off from 7 am to 7 pm) (Metten et al., 2011), and animals were allowed 1 week to acclimate to the reverse light cycle procedure. The BRL50481 injection was performed 1 hour after the lights were turned off, and animals were given a bottle containing 20% EtOH (v/v) 3 hours after the lights were turned off. The EtOH bottle was left in the chamber for 4 hours for 4 consecutive days before the EtOH intake was recorded.

### EtOH 2-Bottle Choice (2BC) Test

EtOH consumption was measured using the 24-hour continuous 2BC paradigm (Barkley-Levenson and Crabbe, 2015). Mice were individually housed in standard plastic cages with 2 plastic bottles placed on wire lids, with each containing a metal tube. Mice had ad libitum access to EtOH or water. The bottles were weighed daily at 5 pm to determine the EtOH consumption.

Mice were given a 1-week 2BC adaptation period and observed for position preference, with EtOH concentrations ranging from 3%, 6%, to 10% (v/v). Each EtOH concentration was administered for 4 consecutive days. A fresh EtOH solution was provided every other day. BRL50481 (5 mg/kg, i.p.) was administered from day 13 to day 19. EtOH preference was calculated as the EtOH intake divided by the total fluid intake (i.e., EtOH and water intake).

### Sucrose or Quinine DID Test

To determine whether the drug effect on EtOH intake was specific, we tested sucrose intake using DID with some modifications (Jimenez Chavez et al., 2021; Arnold et al., 2023). In the sucrose drinking test, mice were given 1 bottle of a sucrose solution (1%, v/v) and another bottle of water for 3 consecutive days after 3 hours of darkness. In the quinine drinking test, mice were given 1 bottle of quinine solution (0.1 mM) and another bottle of water for 3 consecutive days after 3 hours of darkness. The average daily intake of sucrose was calculated using the same procedure as that of EtOH in the 2BC.

### Western-Blot Analysis (WB)

Brain tissues (15 mg) were lysed using an radioimmunoprecipitation assay (RIPA) buffer containing protease and phosphatase inhibitors (Solarbio, China). Proteins (20 μg) were analyzed by sodium dodecyl sulfate (SDS)-polyacrylamide electrophoresis using the gel electrophoresis system (Bio-Rad, Hercules, California, USA). Enhanced chemiluminescence detection reagent (Thermo Scientific, Massachusetts, Waltham, USA) was used to observe the immunoreactivity bands. Protein quantification was performed using the BCA kit (Solarbio). The antibodies against PDE1B (Cat# ab182565, 1:1000), PDE2A (Cat# ab140650, 1:1000), PDE4A (Cat# ab200383, 1:1000), PDE4B (Cat# ab170939, 1:1000), PDE4D (Cat# ab171750, 1:1000), PDE7B (Cat# ab170914, 1:1000), PDE10A (Cat# ab227829, 1:1000), or PKA (Cat# ab32514, 1:1000) were purchased from Abcam (Cambridge, UK). The PDE7A antibody (Cat# sc-398031, 1:500) was purchased from Santa Cruz Biotechnology (Texas, USA). The antibodies against phospho-CREB (ser133, Cat# 9198, 1:1000), CREB (Cat# 9197, 1:1000), phospho-PKA (Thr197, Cat# 5661, 1:1000), Epac2 (Cat# 4156, 1:1000), phospho-p44/42 mitogen activated protein kinase (MAPK) (Erk1/2) (Thr202/Tyr204, Cat# 4370, 1:1000), or p44/42 MAPK (Erk1/2) (Cat# 4695, 1:1000) were purchased from Cell Signaling Technology, Inc (Danvers, Massachusetts, USA). Theβ-actin (Cat# TA-09, 1:2000) and glyceraldehyde-3-phosphate dehydrogenase (GAPDH) (Cat# TA-08, 1:2000) antibodies were purchased from Zhong Shan Biology (ZSbio, Beijing, China). The ratio of target protein to the respective reference protein in each lane was calculated for subsequent statistical analysis.

### cAMP ELISA (Enzyme-Linked Immunosorbnent Assay)

After homogenizing, the brain tissues were centrifuged at 3000 ×*g* for 10 minutes. The supernatant was taken and assayed using the cAMP ELISA kit (Shanghai ELISA, China); all samples were tested in duplicates and compared with the standards provided in the kit. The absorbance of the samples was obtained using a 96-well microplate at a wavelength of 450 nm and processed using the SkanIt RE 6.1.1 software (Thermo Scientific) that came with the kit. The cAMP levels were normalized to protein content and expressed as pmol/mg protein.

### BEC Measurement

Blood was collected from the neck after decapitation or from tail to detect BECs. The serum was collected after centrifugation of the blood according to the Anolox blood Alcohol Detector (UK) instructions, and BECs were measured in mg/dL using a custom EtOH detection reagent.

### Materials and Drugs

The EtOH solutions were prepared using absolute EtOH (v/v) (Kermel, China) in double distilled water. BRL50481, purchased from MCE (Cat# HY-109586, Monmouth Junction, NJ, USA), was dissolved in saline containing 1% dimethyl sulfoxide (DMSO) (Cat# PWL064, Mei Lun, China). The cAMP ELISA kit (Cat# ml076939) was purchased from Shanghai Enzyme-linked Biotechnology Co., Ltd. All drug solutions were freshly prepared before testing.

### Data Analysis

Statistical analysis was performed by Prism V8.0 software (GraphPad Software, Inc.). Wilcoxon signed-rank test was used for 2 paired groups analysis. Mann–Whitney U test was performed for 2 unpaired groups analyses. For more than 3 unpaired groups analyses, 1-way ANOVA followed by Holm–Sidak’s multiple comparison test was used. Only significant comparisons were labeled in the figures. Lines and error bars in all figure dot plots indicate mean and SEM. Only significant comparisons were labeled in the figures. The level of significance was set at *P < *.05.

## RESULTS

### Female Mice Exhibited Pronounced Stimulatory Effects, Minimal Sedative Effects, and Heightened Ethanol Sensitivity

**Figure 1.**
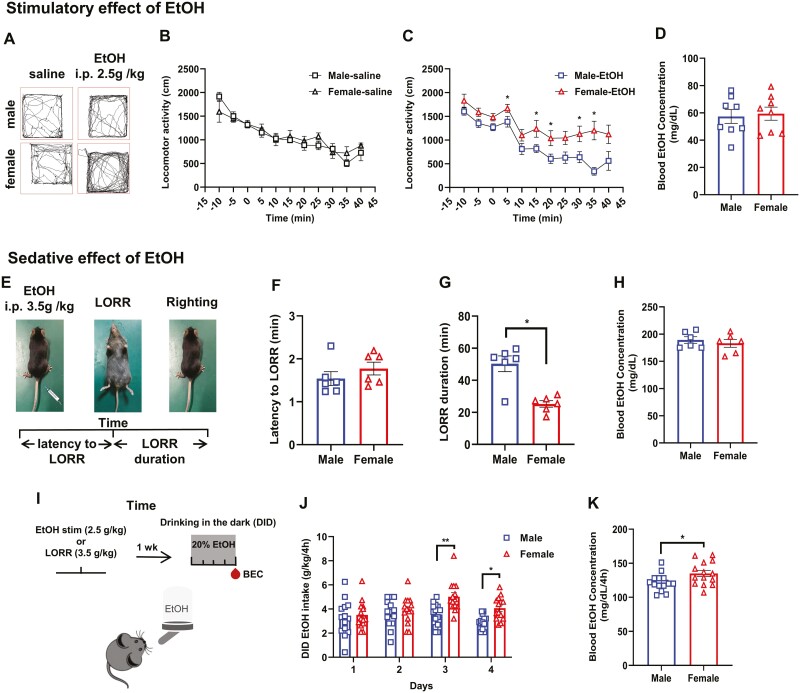
Female mice exhibited pronounced stimulatory effects, minimal sedative effects, and heightened ethanol sensitivity. (A) Sample traces of locomotor activity for 5 minutes in C57BL/6J female and male mice following saline or ethanol (EtOH) (i.p., 2.5g/kg) injection. (B) The locomotor activity of the male and female mice was counted at 5-minute intervals, with a saline injection administered 15 minutes before baseline measurement and 40 minutes following (*t* test, *P* > .05, N = 8 for each group). (C) Locomotor activity of C57BL/6J female and male mice at 15 minutes before EtOH injection (i.p., 2.5g/kg) and 40 minutes following (*t* test, *P < .05; N = 8 for each group). (D) Blood ethanol concentration (BEC) of male and female mice at 2 hours after EtOH injection (*t* test, *P *=* *.9591, N = 8 for each group). (E) Schematic illustrating the loss of righting reflex (LORR) test. (F) Time taken to enter the LORR after ethanol injection (i.p., 3.5g/kg) in C57BL/6J female and male mice (*t* test, *P *=* *.3268, N = 6 for each group). (G) Time taken to recover the righting reflex (*t* test, *P *=* *.0152, N = 6 for each group). (H) BEC of male and female mice at 90 minutes after injection of EtOH (*t* test, *P *=* *.6753, N = 6 for each group). (I) Time of DID protocol showing sequence of tests and days in which blood samples (red drop) were taken and processed for BEC. (J) Consumption of 20% EtOH in male and female mice of the DID experiment (*t* test, day = 3, 4; *P < .05, **P < .01, N = 14 for each group). (K) BEC of male and female mice after the DID experiment (*t* test, *P *=* *.0317, N = 14 for each group). All data shown are means ± SEM.

Previous studies have revealed sex differences in EtOH consumption (Yoneyama et al., 2008; Blegen et al., 2018). In the present study, we conducted a comprehensive investigation into the sex-specific effects of EtOH administration in mice. Initially, we characterized and validated the locomotor measurements as a reliable method for assessing the biphasic stimulatory and sedative effects induced by EtOH in both male and female mice. All mice were individually placed in separate chambers to assess for the locomotor activity following of EtOH or saline injections (Figure 1A). As illustrated in Figure 1B, the differences in locomotor activity between the male and female mice were not discernible following saline injection. However, upon EtOH administration, the female mice exhibited significantly elevated levels of locomotion compared with male mice, indicating a heightened sensitivity to EtOH stimulation (Figure 1C). After a 2-hour period following EtOH injection, blood samples were collected from the tail for BEC measurement. The results revealed no significant disparity in BEC between the male and female mice (Figure 1D).

Next, the LORR test was conducted to validate the sedative effects of EtOH (Figure 1E). The results showed that the duration of the righting reflex recovery, also known as LORR duration, was significantly shorter in female mice than in male mice, whereas no significant sex difference was observed in the latency to LORR (Figure 1F, G), indicating that female mice exhibit greater resistance to EtOH-induced sedation. Additionally, Figure 1H illustrates comparable blood EtOH concentrations between sexes.

Given the observed heightened stimulatory and diminished sedation effects of EtOH in female mice, we hypothesized that female mice would exhibit a greater proclivity toward voluntary EtOH consumption. Subsequently, all mice were allowed to freely access the 20% EtOH solution in their home cages for 4 hours per day for 4 successive days. This voluntary drinking paradigm was adapted from the DID paradigm (Figure 1I). Consistent with the enhanced stimulation, female mice consumed more EtOH than male mice during DID (Figure 1J). The neck blood samples were taken on the fourth day for the BEC assay. There was also a higher BEC following voluntary drinking in the female mice (Figure 1K).

Collectively, these data suggest that, in female mice, the pronounced stimulatory effects and reduced sedative properties of EtOH, along with its heightened sensitivity, hold prognostic significance for alcohol abuse.

### PDE7A Expression Was Upregulated Following Ethanol Administration in Female Mice

**Figure 2.**
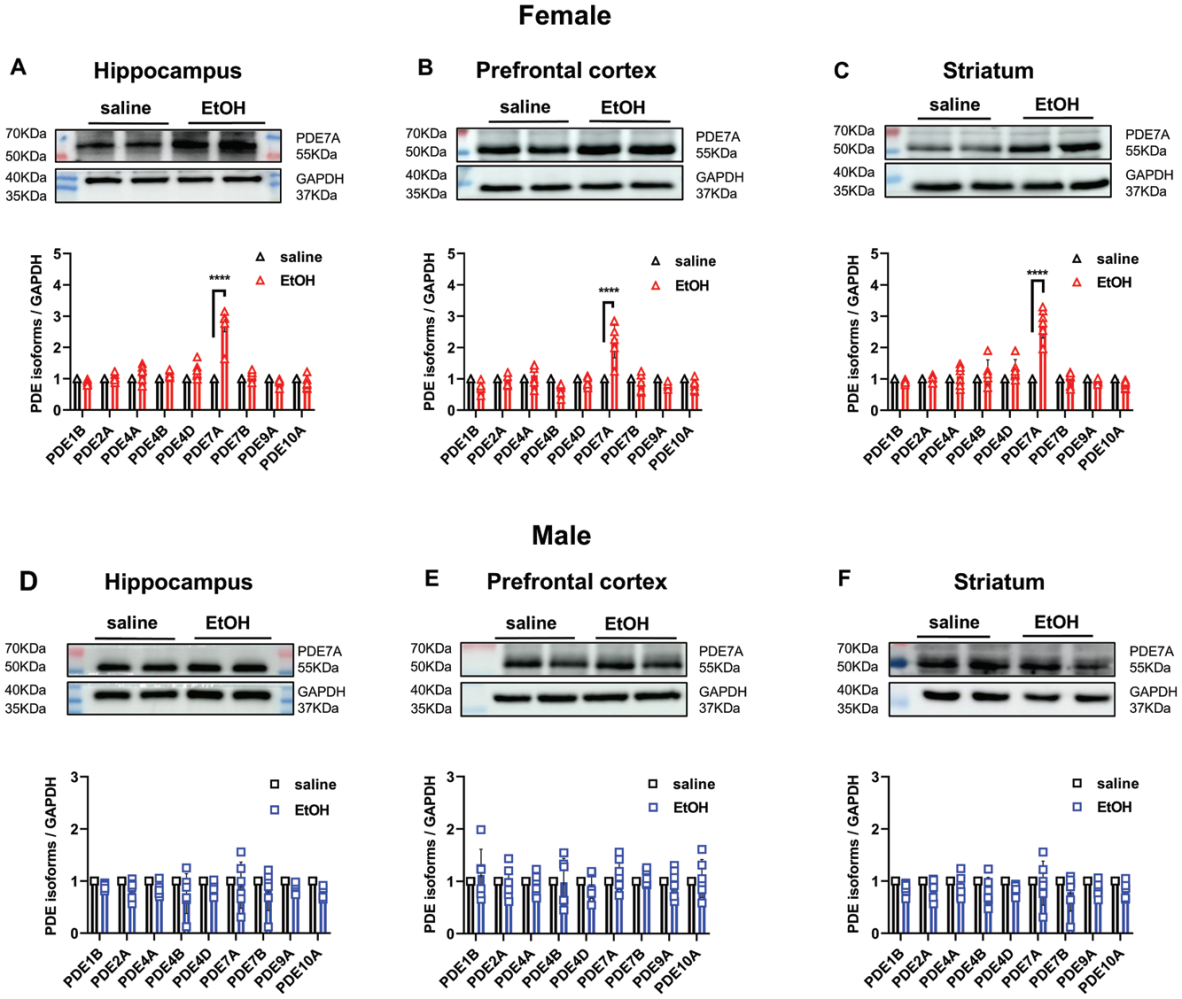
PDE7A expression was upregulated following ethanol administration in female mice. (A–C) Protein expression of PDE isoforms, including PDE1B, PDE2A, PDE4A, PDE4B, PDE4D, PDE7A, PDE7B, PDE9A, and PDE10A, in the hippocampus, prefrontal cortex, and striatum of C57BL/6J female mice after saline or EtOH (i.p., 2.5 g/kg) injection for 7 days. The upper image shows the true bands of PDE7A and GAPDH at their respective KDa quantitative value (*t* test, PDE7A: EtOH vs saline, ****P < .0001). (D–F) Expression of PDE isoforms in male mice after EtOH injection. The upper image shows the true bands of PDE7A and GAPDH at their respective KDa quantitative value (*t* test, PDE7A: EtOH vs saline, *P* > .05). N = 6 mice for each group. All data shown are means ± SEM. The ratio of PDE7A to the respective GAPDH in each lane was calculated for statistical analysis.

To elicit a transient and rapid increase in BECs and selectively target the PDE subtypes that are highly associated with EtOH, i.p. injections of EtOH (2.5 g/kg) were performed once daily for 7 consecutive days. The selected PDE subtypes, including PDE1B, PDE2A, PDE4A, PDE4B, PDE4D, PDE7A, PDE7B, PDE9A, and PDE10A, which exhibit a more widespread distribution in the brain, were investigated. A significant upregulation of the hippocampal expression of the specific subtype PDE7A was observed at 2 hours after EtOH injections in female mice, whereas no such effect was observed in male mice (Figure 2A, D). Similarly, elevated PDE7A levels in the prefrontal cortex and striatum were exclusively observed in female mice (Figure 2B and E and 2C and F, respectively).

To demonstrate that the observed disparity was not attributable to the heightened PDE7A expression in female mice, we assessed the basal level of PDE7A expression in 8-week-old mice. Intriguingly, our data showed a consistently lower basal level of PDE7A in the female mice than in the male mice across all the 3 brain regions (supplemental Fig. 1A–C).

### Transgenic Systemic Knockdown PDE7A Attenuated Sex Disparity in Ethanol Responsiveness

**Figure 3.**
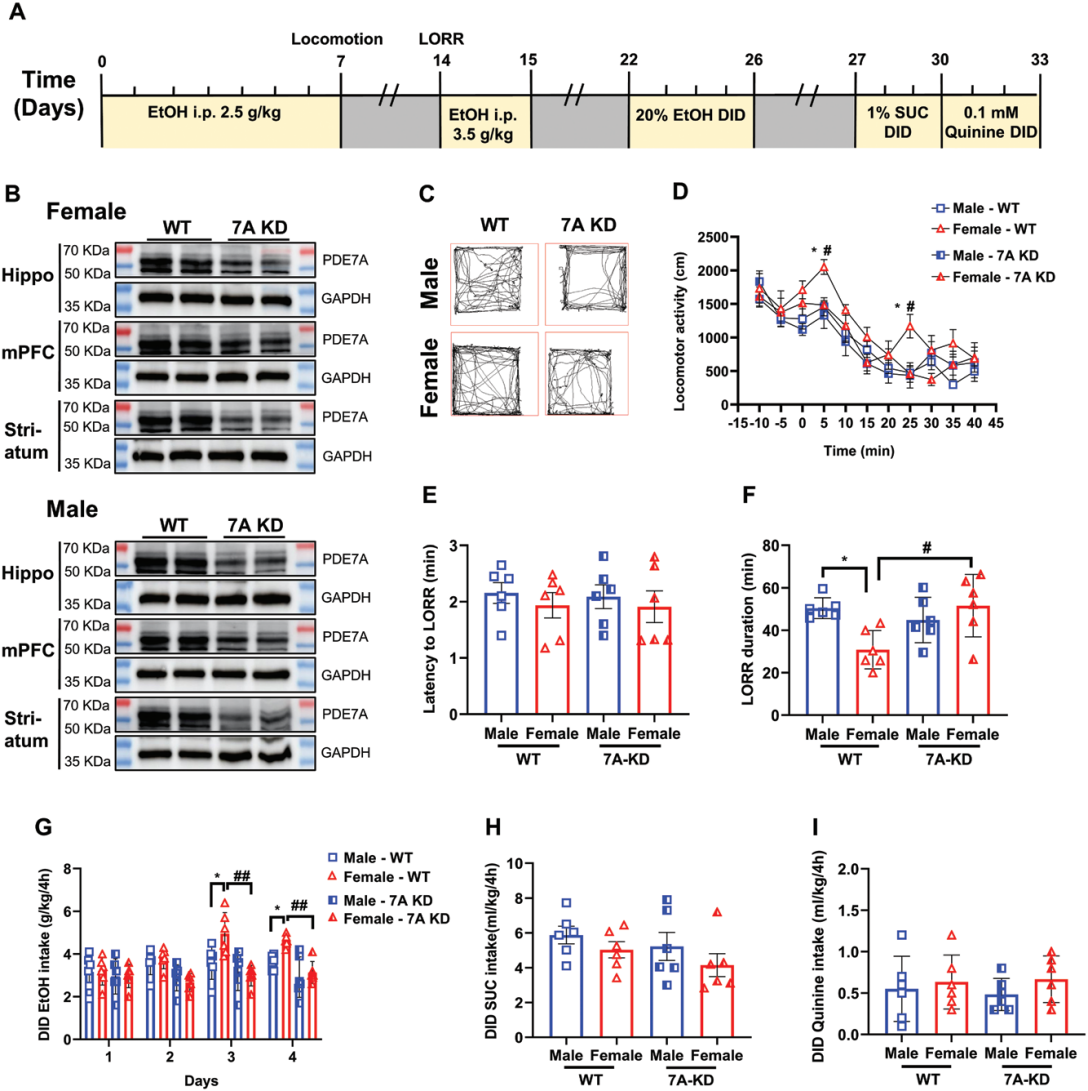
Transgenic systemic knockdown of PDE7A attenuated sex disparity in ethanol responsiveness. (A) Timeline of PDE7A knockdown (7A KD) mice throughout the experiment. The yellow parts represent the experimental time and the gray parts represent the recovery time of mice. (B) The efficiency of PDE7A knockdown in the hippocampus, prefrontal cortex, and striatum of both male and female mice. (C) The trajectories of female and male WT mice and 7A KD mice at 5 minutes after EtOH injection (i.p., 2.5 g/kg). (D) The locomotor activity was counted at 5-minute intervals between female and male WT mice or 7A KD mice (1-way ANOVA, time = 5, 25 minutes; F_(3,20)_ = 0.88, 0.68; female-WT vs male-WT, *P < .05, female-WT vs female-7A KD, ^#^*P* < .05). (E–F) The latency to loss of the righting reflex and the time taken to the recovery the righting reflex in male and female WT mice or 7A KD mice after EtOH injection (i.p., 3.5 g/kg) (1-way ANOVA, latency to LORR: F_(3,20)_ = 0.63, *P* > .05; LORR duration: F_(3,20)_ = 1.25, female-WT vs male-WT, *P < .05; female-WT vs female-7A KD, ^#^*P* < .05). (G) The consumption for 20% EtOH in male and female WT mice or 7A KD mice (1-way ANOVA, day = 3,4; F_(3,20)_ = 1.08, 1.77; female-WT vs male-WT, *P < .05; female-WT vs female-7A KD, ^##^*P* < .01). (H) The intake of 1% sucrose solution in male and female WT as well as PDE7A-KD mice (1-way ANOVA, F_(3,20)_ = 0.63, *P* > .05). (I) The intake for 0.1 mM quinine solution in male and female WT and PDE7A-KD mice (1-way ANOVA, F_(3,20)_ = 0.70, *P* > .05). N = 6 mice for each group. All data shown are means ± SEM.

To further investigate the role of PDE7A in regulating the EtOH-induced effects in male and female mice, we employed transgenic mice with PDE7A knockdown (referred to as 7A KD). Figure 3A illustrates the complete timeline of EtOH sensitivity, stimulation, and sedation observed in the PDE7A knockdown mice. WB analysis was conducted to assess the efficiency of PDE7A knockdown in the hippocampus, prefrontal cortex, and striatum of both male and female mice (Figure 3B), which the statistics of knockdown efficiency of PDE7A is shown in supplemental Fig. 2A and B. Following the 7-day EtOH injections, there were no significant differences observed in the locomotor activity between the male and female 7A KD mice within the initial 5 minutes. Furthermore, a significant reduction in EtOH-induced locomotor activity, specifically in female mice upon knockdown of PDE7A, was observed, whereas no such effect was observed in the male mice (Figure 3C, D). We also observed that the latency to LORR remained unchanged regardless of sex and genotype differences (Figure 3E). However, the LORR duration was reduced in female mice; this difference disappeared following PDE7A knockdown (Figure 3F). Furthermore, PDE7A knockdown resulted in a significant increase in LORR duration, particularly among female mice (Figure 3F), which exclusively led to a notable decrease in EtOH consumption specifically among female mice (Figure 3G). It is worth noting that PDE7A knockdown did not exert any influence on taste preference, including sucrose or quinine consumption in both male and female mice (Figure 3H, I), thereby indicating that PDE7A selectively modulates neuro-addictive substances, such as alcohol, rather than interferes with innate rewarding stimuli such as sucrose.

### Pharmacological Inhibition of PDE7A Attenuates Ethanol Responsiveness and Consumption in Female Mice

EtOH elicits an initial rapid stimulatory effect followed by a prolonged sedative response, which may serve as potential predictors of EtOH consumption (Bocarsly, 2019). To further investigate the role of PDE7A in regulating the EtOH-induced behavioral responses in female mice, we employed BRL50481, a selective inhibitor of PDE7 (IC50 values of 0.15 and 12.1 μM for PDE7A and PDE7B, respectively). Briefly, BRL50481 (5 mg/kg, i.p.) was administered 2 hours before the EtOH injection for 7 consecutive days, and locomotor activity was assessed on the final day. To ensure its efficacy in inhibiting PDE7, an i.p. dose of 5 mg/kg was administered, with minimal occurrence of vomiting-related side effects (Martinez and Gil, 2014).

**Figure 4:**
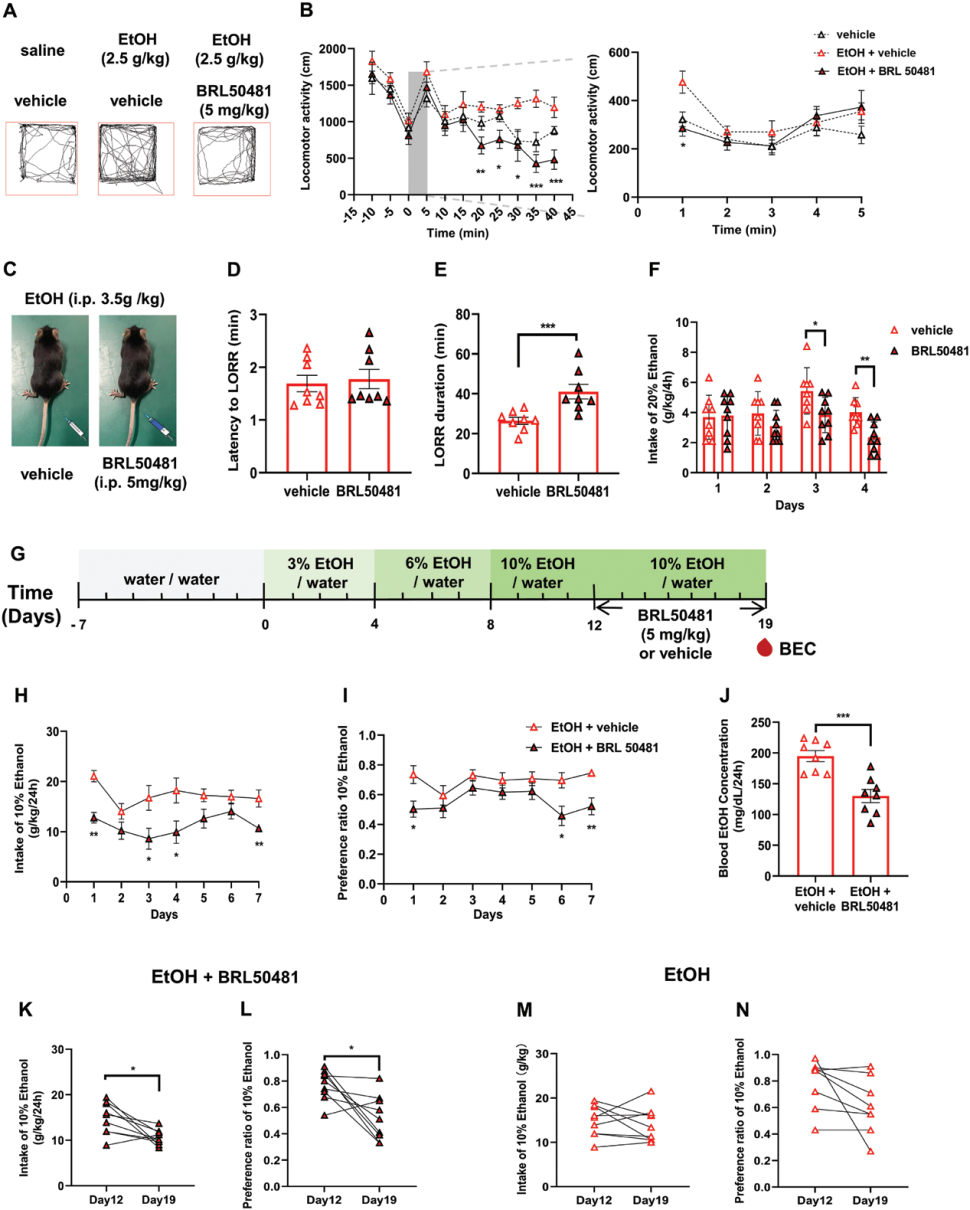
Pharmacological inhibition of PDE7A attenuated ethanol responsiveness and consumption in female mice. (A) The locomotor trajectories of female mice were recorded at 5 minutes after i.p. injection of saline or EtOH or EtOH (i.p. 2.5g/kg) with BRL50481 (i.p. 5 mg/kg). (B) Left, locomotor activity of C57BL/6J female mice for 15 minutes before injecting saline or EtOH or EtOH with BRL50481 and 40 minutes after. The shaded box highlights that the locomotor activity is required to be analyzed by minutes (1-way ANOVA, time = 20, 25, 30, 35, and 40; F_(2,21)_ = 0.64, 1.65, 5.65, 0.06, and 4.91; *P < .05, **P < .01, ***P < .001). Right, locomotor activity of female mice analyzed in minute (1-way ANOVA, F_(2,21)_ = 1.10, *P = *.0379). (C) BRL50481 was administered at a dose of 5 mg/kg at 2 hours before EtOHl injection (i.p., 3.5 g/kg). (D) Time required for the loss of righting reflex following administration of EtOH or EtOH combined with BRL50481 in female mice (*t* test, *P* > .05). (E) Time required for the recovery of righting reflex following administration of EtOH or EtOH combined with BRL50481 in female mice (*t* test, *P = *.0152). (F) Consumption of 20% EtOH in male and female mice of the DID experiment (*t* test, day = 3,4; *P < .05, **P < .01). (G) 2BC paradigm used to examine the effect of BRL50481 on EtOH consumption and preference rate in female mice. (H–I) The daily EtOH intake and preference rate for 10% EtOH in female mice treated with BRL50481 over a period of 7 days (*t* test, *P < .05, **P < .01). (J) The BEC for 10% ethanol on the seventh day of injecting BRL50481 in C57BL/6J female mice (*t* test, *P = *.0009). (K–L) Paired *t* test for analyzing EtOH intake and preference rate after BRL50481 administration for 7 days in female mice (Wilcoxon test, *P < .05). (M–N) Paired *t* test to analyze EtOH intake and preference rate in female mice administrated with the vehicle (Wilcoxon test, *P* > .05). N = 8 mice for each group. All data shown are means ± SEM.

We found a significant reduction in EtOH-induced locomotor activity specifically in the female mice upon BRL50481 administration (Figure 4A and B), whereas no such effect was observed in the male mice (supplemental Fig. 3A and B). Additionally, the inhibition of PDE7A by BRL50481 produced a significant increase in LORR duration only in the female mice (Figure 4C and E), whereas no effect was observed in the male mice (supplemental Fig. 3C and E). Furthermore, BRL50481 resulted in a significant reduction in EtOH consumption in the DID test in the female mice (Figure 4F), whereas no changes were observed in the male mice (supplemental Fig. 3F). Similar to the PDE7A-KD mice, BRL50481 did not exert any influence on sucrose or quinine consumption in either male and female mice (supplemental Fig. 3G–J). These results demonstrated that PDE7A inhibition attenuated the EtOH-induced stimulation, sedation resistance, and sensitivity simply in female mice, indicating a possible involvement of PDE7 in modulating the alcohol consumption specifically without impact on the taste.

The greater resistance of C57 mice to EtOH-induced stimulatory and sedative effects is directly proportional to the amount of autonomous EtOH consumed by the mice at later stages (Bocarsly, 2019). Based on the results showing that PDE7A inhibition modulated the responsiveness of female mice to EtOH, we used the 2BC paradigm to examine the effect of BRL50481 on EtOH consumption and preference rate in female mice (Figure 4G). To ensure better adaptation to drinking, the EtOH concentrations were set at 3%, 6%, and 10% based on a previous study (Crabbe and Weigel, 1987). As shown in Figure 4H and I, the daily EtOH intake and preference rate of female mice treated with BRL50481 for 7 days were significantly decreased.

Furthermore, a paired *t* test was employed to analyze EtOH intake and preference rate before and after BRL50481 administration. Notably, the administration of BRL50481 on the seventh day significantly reduced female alcohol intake and preference rate (Figure 4J and K). Consistently, the neck blood samples collected from female mice also showed a significant decrease in BEC following BRL50481 administration (Figure 4L).

### PDE7A Inhibitor–Regulated Drinking Behavior May Be Associated With cAMP-PKA/Epac2 Pathways and Levels of CREB and ERK

**Figure 5.**
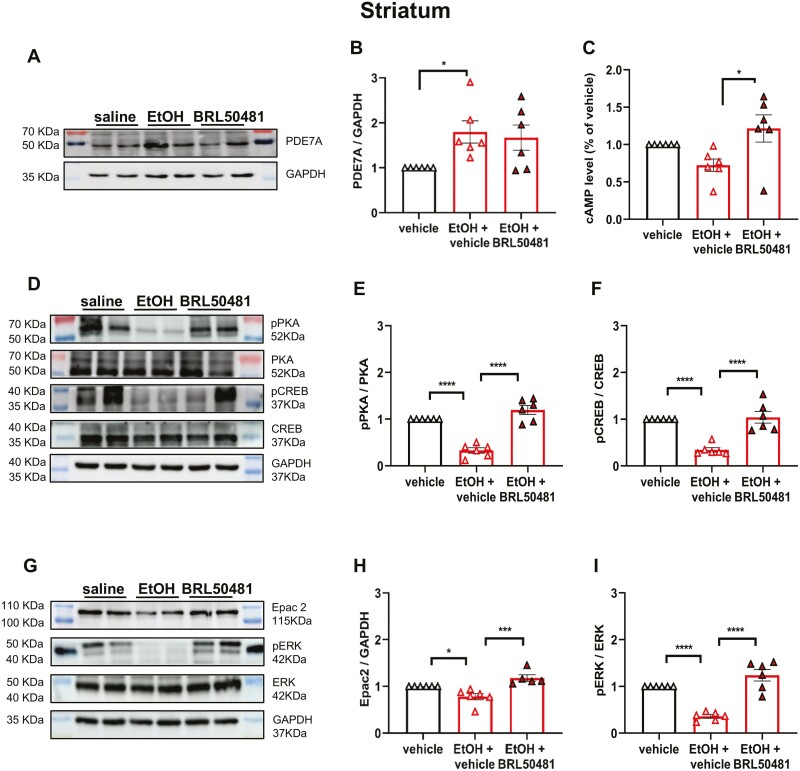
PDE7A inhibitor regulated the drinking behavior by the cAMP-PKA/Epac2 pathways and downstream phosphorylation of CREB and ERK. (A–B) The protein expression level of PDE7A in the striatum of female mice was quantified via western-blot analysis following the 2BC test (1-way ANOVA, F_(2,12)_ = 5.16, *P < .05). (C) ELISA was used to measure the cAMP level in the striatum (1-way ANOVA, F_(2,12)_ = 6.16, *P < .05). (D–F) The protein levels of pPKA, PKA, pCREB, and CREB in the striatum of female mice were quantified following exposure to ethanol and treatment with BRL50481 (1-way ANOVA, pPKA/PKA, F_(2,12)_ = 6.36; pCREB/CREB, F_(2,12)_ = 6.98; ****P < .0001). (G–I) The protein levels of Epac2, pERK, and ERK in the striatum of female mice were quantified following exposure to EtOH and treatment with BRL50481 (1-way ANOVA, Epac2, F_(2,12)_ = 1.69; pERK/ERK, F_(2,12)_ = 7.4; *P < .05, ****P < .0001). N = 6 mice for each group. All data shown are means ± SEM, the ratio of PDE7A to the respective GAPDH in each lane was calculated for statistical analysis.

To further investigate the underlying mechanism of PDE7A in regulating EtOH drinking behavior, cAMP signaling was tested after the 2BC drinking paradigm. The protein expression of PDE7A in the striatum of female mice is significantly upregulated following EtOH consumption (Figure 5A and B). Additionally, as illustrated in Figure 5C, ELISA analysis demonstrated a significant reduction in cAMP levels following EtOH consumption; however, this effect was reversed upon BRL50481administration. Contrarily, the pPKA, PKA, pCREB, and CREB protein levels in the striatum of female mice were assessed using WB analysis. As shown in Figure 5E, the ratios of pPKA to PKA and pCREB to CREB in the striatum were significantly decreased after EtOH exposure in 2BC, which was effectively reversed following BRL50481 treatment (Figure 5D–F). The cAMP-Epac2 activation in both the peripheral (gut) and CNS has been previously demonstrated to significantly enhance the phosphorylation level of downstream ERK (Lotfi et al., 2006; Portugal et al., 2019). Previous studies have demonstrated that ethanol self-administration can be regulated by inhibiting pERK1/2 (Faccidomo et al., 2009), indicating the involvement of multiple molecular pathways in EtOH consumption. Therefore, we examined the protein expression of Epac2, pERK, and ERK. Intriguingly, after EtOH intake with 2BC, both Epac2 and the ratio of pERK to ERK were decreased, which was reversed by BRL50481 administration (Figure 5G–I).

In addition to the striatum, 2 other brain regions, the prefrontal cortex and hippocampus, were also investigated. Similarly, consistent with the observations in the striatum, PDE7A was increased, and cAMP tended to decrease following 2BC; these were reversed by BRL50481 administration (supplemental Figure 4A, B, H, I, J, and P). Moreover, the ratios of pPKA to PKA, pCREB to CREB, and pERK to ERK, as well as Epac2 expression, exhibited a significant reduction in response to EtOH consumption; however, all these effects were effectively reversed upon BRL50481 administration (supplemental Figure 4A, C–G, I, K–O).

## DISCUSSION

In the present study, we demonstrated for the first time, to our knowledge, that PDE7A is involved in EtOH drinking behavior regulation and sex differences, with the specific results as follows: (1) PDE7A was upregulated in the hippocampus, prefrontal cortex, and striatum in female mice after 7 consecutive days with EtOH injections (i.p.). Contrarily, no such changes were observed in male mice; (2) BRL50481, a selective inhibitor of PDE7, attenuated EtOH responsiveness in stimulation, sedation resistance, and sensitivity exclusively in female mice; (3) PDE7A knockdown with transgenic mice attenuated the sex disparity in EtOH responsiveness; and (4) PDE7A inhibition also reduced the EtOH consumption and preference rate in female mice, which may be associated with the cAMP-PKA/Epac2 pathways and downstream phosphorylation of CREB and ERK. Therefore, the results not only advance our understanding of the role of PDE7A in the responsive effects to EtOH but also underscore the potential of targeting PDE7A as a therapeutic approach for managing alcohol abuse and addiction.

The magnitude of the stimulatory effect induced by EtOH is directly proportional to the subsequent autonomous drinking behavior in mice and even the incidence rate of alcohol abuse and addiction (Hedlund and Wahlström, 2000; Karatayev et al., 2010; Linsenbardt and Boehm, 2012). Conversely, the sedative effect caused by EtOH exhibits an opposite trend (Bocarsly, 2019). Several studies have demonstrated that these 2 parameters are influenced by various factors, including but not limited to age (Quoilin et al., 2013), sex (Quoilin et al., 2010), and individual differences (Barbier et al., 2009; Quoilin et al., 2012). In our study, the female mice exhibited a higher sensitivity to EtOH compared with male mice. Additionally, female mice demonstrated greater resilience against EtOH-induced sedation than male mice, as indicated by the prolonged LORR duration. Furthermore, investigations using the DID paradigm revealed that female mice displayed significantly elevated consumption of 20% EtOH solutions and higher BECs compared with male mice.

PDE4 inhibition reduces EtOH consumption in male mice (Liu et al., 2017; Nentwig et al., 2019); however, none of the abovementioned reports has tested female mice or investigated the potential sex differences. Our study data revealed an immediate upregulation of PDE7A protein levels in the brains of female mice following i.p. injections of EtOH for 7 consecutive days, whereas no such upregulation was observed in male mice. Interestingly, we also found lower baseline levels of PDE7A in 2-month-old female mice than in age-matched male mice, indicating that the rapid mobilization of PDE7A after EtOH administration in female mice was independent of the self-expression levels. These findings further support the strong association between PDE7A and EtOH response, specifically in female mice.

Chronic estradiol replacement has been shown to strengthen the reward mechanism in EtOH drinking by the neurons in the ventral tegmental area in the brain by activating ERα and ERβ, ultimately increasing the ethanol consumption and preference without increasing water consumption in ovariectomized animals (Hilderbrand and Lasek, 2018; Vandegrift et al., 2020). Ovariectomized female mice reportedly exhibited significantly reduced EtOH consumption and preference rates (Becker et al., 1985). PDE7 may affect ovulation by inhibiting the premature synthesis of progesterone and progesterone receptors (Thomas et al., 2002; Vigone et al., 2018), which eventually has an impact on hormone levels. Contrarily, hormones may modulate the PDE7 levels in the brain, although this needs to be further explored in further studies.

We employed transgenic mice with PDE7A knockdown, and our results indicate that PDE7A downregulation partially mitigates the sex-related differences observed in EtOH-induced stimulation, sedation, and sensitivity in mice. To further explore the underlying mechanism, we employed BRL50481 to inhibit the PDE7A function and observed the following effects in female mice: (1) reduced sensitivity to EtOH; (2) diminished stimulation by EtOH; and (3) decreased resistance to EtOH’s sedative properties.

Although these findings were conducted in C57 male mice, targeting PDE7A to specifically regulate the EtOH reactivity in female mice may offer a more effective approach compared with non–sex-specific target for treating AUD in female mice, which is in line with prior results indicating the involvement of the cAMP-PKA pathway in responsiveness to ethanol (Asyyed et al., 2006). EtOH-induced sensitization is associated with increased pCREB levels in mice and decreased brain-derived neurotrophic factor (BDNF) levels, a downstream target of CREB, in the nonsensitized group (Nona et al., 2013). Knockdown of PDE7A may potentially enhance the in vivo cAMP levels following prolonged EtOH administration, thereby partially reversing the biphasic effect on EtOH production. Although sex differences could arise from a combination of factors, including hormones and stress, it is undeniable that PDE7A exert a possible association in this aspect to some extent.

Short-term or acute EtOH exposure increases voluntary EtOH intake (Bocarsly, 2019). Therefore, longer independent drinking experiments are necessary to establish the efficacy of BRL50481 on EtOH intake and preference rates. We conducted a comparative analysis of EtOH consumption and preference rates in mice before and after drug treatment, revealing that BRL50481 specifically reduced both EtOH consumption and preference rates exclusively in female mice. We examined the cAMP pathway and found a significant decrease in cAMP-PKA-pCREB/CREB signaling molecules in the brain of female mice after EtOH consumption. This was supported by previously reported findings demonstrating that increasing cAMP-PKA transduction reduces EtOH consumption in mice (Misra and Pandey, 2006) and chronic EtOH intake also decreases CREB phosphorylation (Pandey et al., 2005).

Growing evidence shows that increasing cAMP-PKA transduction can reduce EtOH consumption in mice. The injections of PKA agonists (Sp-cAMPS) into the amygdala reduce EtOH consumption in alcohol-preferred rats, whereas the injections of PKA inhibitors (Rp-cAMPs) into the nucleus accumbens in the striatum increase EtOH consumption (Beninger and Gerdjikov, 2004; Pandey et al., 2005). The injections of exogenous BDNF into the striatum or neuropeptide Y (NPY) into the amygdala (Robinson et al., 2023) also reduce the EtOH consumption in mice (Jeanblanc et al., 2009). Our data show that BRL50481 can increase the cAMP levels and protein expression of PKA-pCREB/CREB in the brain of female mice after EtOH consumption, suggesting that BRL50481 may reverse the EtOH-induced molecular changes in the brain associating with the cAMP-PKA pathway.

It has been proven that, although the cAMP activated exchange proteins, the Epac2 family is commonly used to enhance the long-term memory of long-term potentiation (LTP) (Naz et al., 2022), microvascular endothelium (Ikeda et al., 2021), and heart disease studies (Fujita et al., 2017). Although Epac2 and PKA have different functions, they share the cyclic nucleotide binding of cAMP (Robichaux and Cheng, 2018). Epac2 stimulates downstream rap1, mediates extracellular signal-regulated kinase activation, and simultaneously inhibits PKA and ras (Enserink et al., 2002). pERK expression is upregulated following acute EtOH administration (Rosas et al., 2014). However, our study results have revealed a downregulation of ERK phosphorylation in mice exposed to 2BC EtOH consumption. These findings are consistent with the results of a previous study demonstrating that chronic EtOH intake (6%) for 10, 20, or 30 days decreased the pERK content in the striatum in rats. Additionally, the pERK levels rapidly increase following acute EtOH withdrawal (Cui et al., 2011). Generally, it is plausible to consider that the duration of EtOH exposure may potentially exert an influence on ERK phosphorylation; thus, these findings are not contradictory.

We observed a reduction in Epac2 levels within the brains of female mice following EtOH consumption, which was subsequently restored by BRL50481 treatment. These findings suggest that cAMP signaling modulation by BRL50481 administration may not solely result from downstream PKA activation, as alternative downstream signaling cascades may also be involved. This intriguing phenomenon warrants further investigations.

### Strengths and Limitations

The strength of this study lies in the abnormal increase of PDE7A in multiple brain regions following heavy administration of EtOH in female mice, indicating a strong correlation between PDE7A and EtOH drinking behavior. Inhibition of PDE7A expression through various methods was found to significantly impact EtOH responsiveness and subsequent consumption in female mice.

This study has several limitations. First, we did not conduct genomic data mining to explore the potential explanation for the sex-specific expression of PDE7A in AUD. Second, although we used a commercially available and reliable selective inhibitor (BRL50481) to target PDE7A, it may have limited our focus on specific subtypes of PDE7 in AUD. Furthermore, considering the widespread expression of PDE7 in various tissues (Han et al., 1997; Jackson et al., 2007; Johansson et al., 2012), including the brain, skeletal muscle, heart, and kidney, it is necessary to acknowledge that the use of BRL50481 may potentially exert a minimal impact on these sites. Last, although WB analysis did not establish a causal relationship between signaling changes and behavior, it merely indicated their parallel observation. Incorporating additional technical methodologies or employing inhibitors that target specific protein molecules within the pathway could provide further insights into the upstream and downstream relationships in this signaling cascade, thereby offering a more comprehensive understanding of PDE7A’s role in AUD.

## CONCLUSIONS

PDE7A inhibition or knockdown not only exerts sex-specific effects on EtOH-induced stimulation, sedation, and sensitivity in mice but also decreases EtOH consumption in female mice. The changes of behavior by inhibiting of PDE7A may be associated with the cAMP-PKA/Epac2 pathways and downstream phosphorylation of CREB and ERK. These novel findings demonstrate the essential role of PDE7A in regulating EtOH drinking behavior and highlight PDE7A as a potential therapeutic target for AUD. Further studies are needed to elucidate the mechanisms underlying the sex differences in PDE7A-mediated alcohol drinking behavior.

## Supplementary Material

pyae032_suppl_Supplementary_Material

## Data Availability

Data that support the present results are available from the corresponding author upon reasonable request.
