# Correction to: Identification of Phosphodiesterase-7A (PDE7A) as a Novel Target for Reducing Ethanol Consumption in Mice

**DOI:** 10.1093/ijnp/pyae049

**Published:** 2024-11-05

This is a correction to: Ran Wei, Fangjiao Zong, Jiahao Dong, Wei Zhao, Fangfang Zhang, Wei Wang, Shuang Zhao, Ziqi Wang, Fang Zhang, Han-Ting Zhang, Identification of Phosphodiesterase-7A (PDE7A) as a Novel Target for Reducing Ethanol Consumption in Mice, *International Journal of Neuropsychopharmacology*, Volume 27, Issue 8, August 2024, https://doi.org/10.1093/ijnp/pyae032

Following article publication, concerns about overlap between Figures 2D and 5D were raised via PubPeer (https://pubpeer.com/publications/C135EE1B112C811BF33537934C82C2).

The authors subsequently contacted the journal and acknowledged that incorrect immuno-blots had been inserted in the originally published versions of not only Figures 2D and 5D but also Figure 5A. The authors have provided revised versions of both Figures 2 and 5, which the editors reviewed. The editors also concur with the authors’ assessment that these errors do not change the overall findings or the conclusions of the study.

The corrected Figure 2 is shown below and has been replaced in the original article.


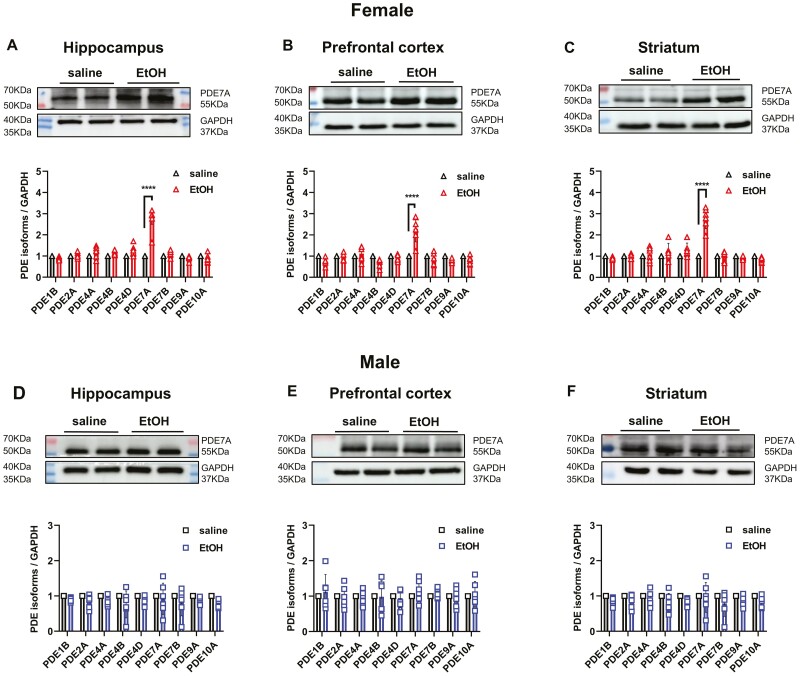


The original, incorrect figure 2 is reproduced below to maintain the scientific record.


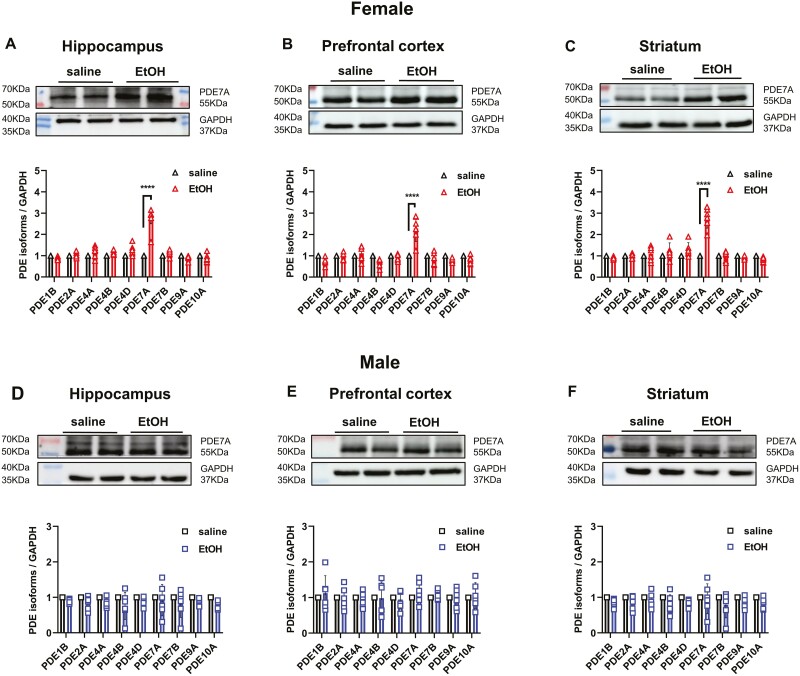


The corrected Figure 5 is shown below and has been replaced in the original article.


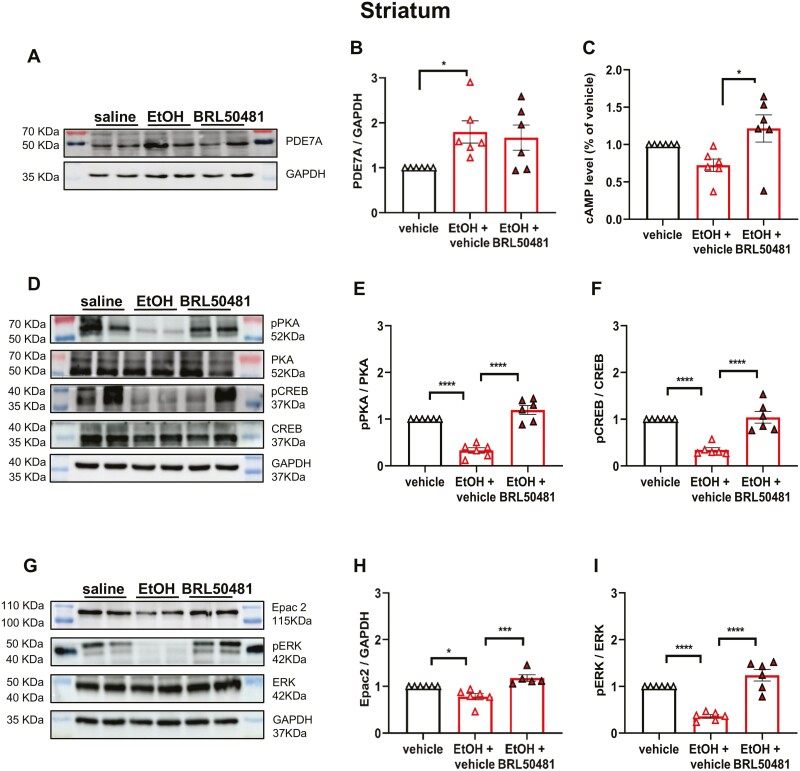


The original, incorrect figure 5 is reproduced below to maintain the scientific record.


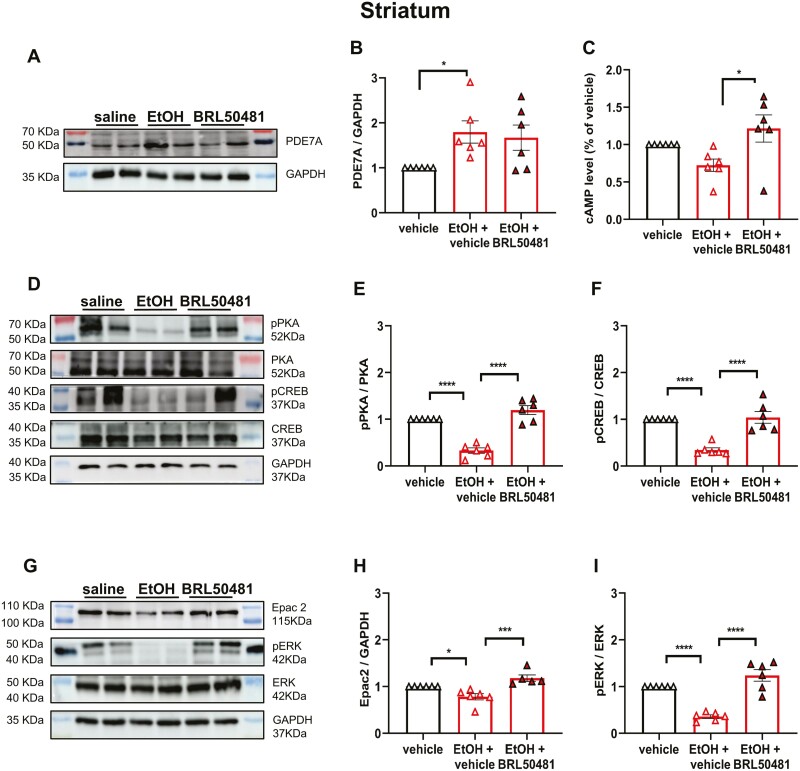


The panels in these figures are now emended in the originally published article.

