# Supplementary material for: Identification of Phosphodiesterase-7A (PDE7A) as a Novel Target for Reducing Ethanol Consumption in Mice
Source: Int J Neuropsychopharmacol. 2024 Aug 5;27(8):pyae032. doi: 10.1093/ijnp/pyae032 (PMC11348009; doi:10.1093/ijnp/pyae032)
Supplement: pyae032_suppl_Supplementary_Material [file pyae032_suppl_supplementary_material.docx]

**Supplementary data**

**Sup Fig1. Female mice have lower basal expression of PDE7A than male mice.**

**
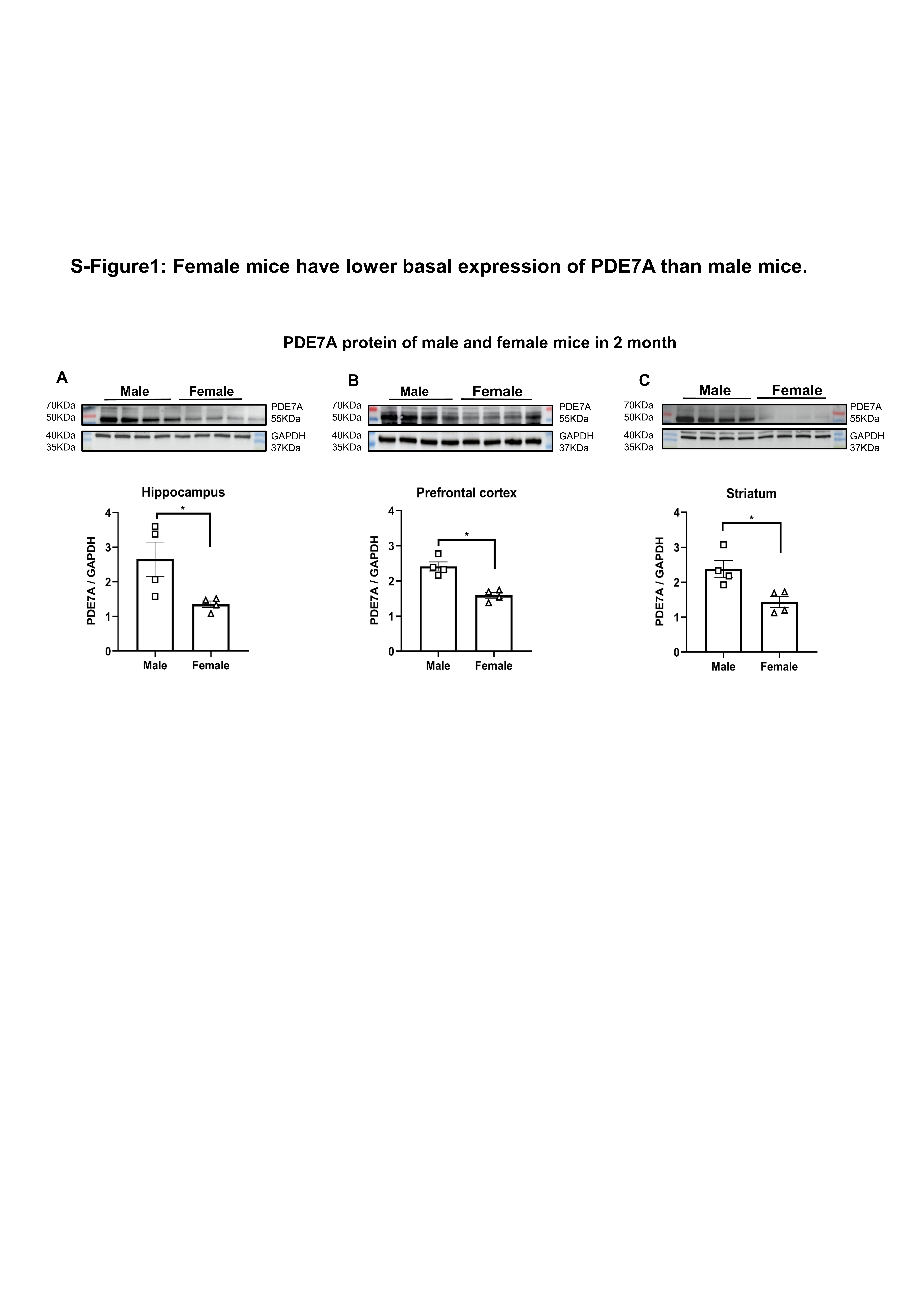
**

Basal expression of PDE7A protein in A) hippocampus, B) prefrontal cortex and C) striatum of 2-month-old male mice and female mice C57BL/6J mice (t-test, *p < 0.05). n = 4 mice for each group. All data shown are means ± SEM, the ratio of PDE7A to the respective GAPDH in each lane was calculated for statistical analysis.

**Sup Fig2. PDE7A is significant knockdown in 7A KD mice.**


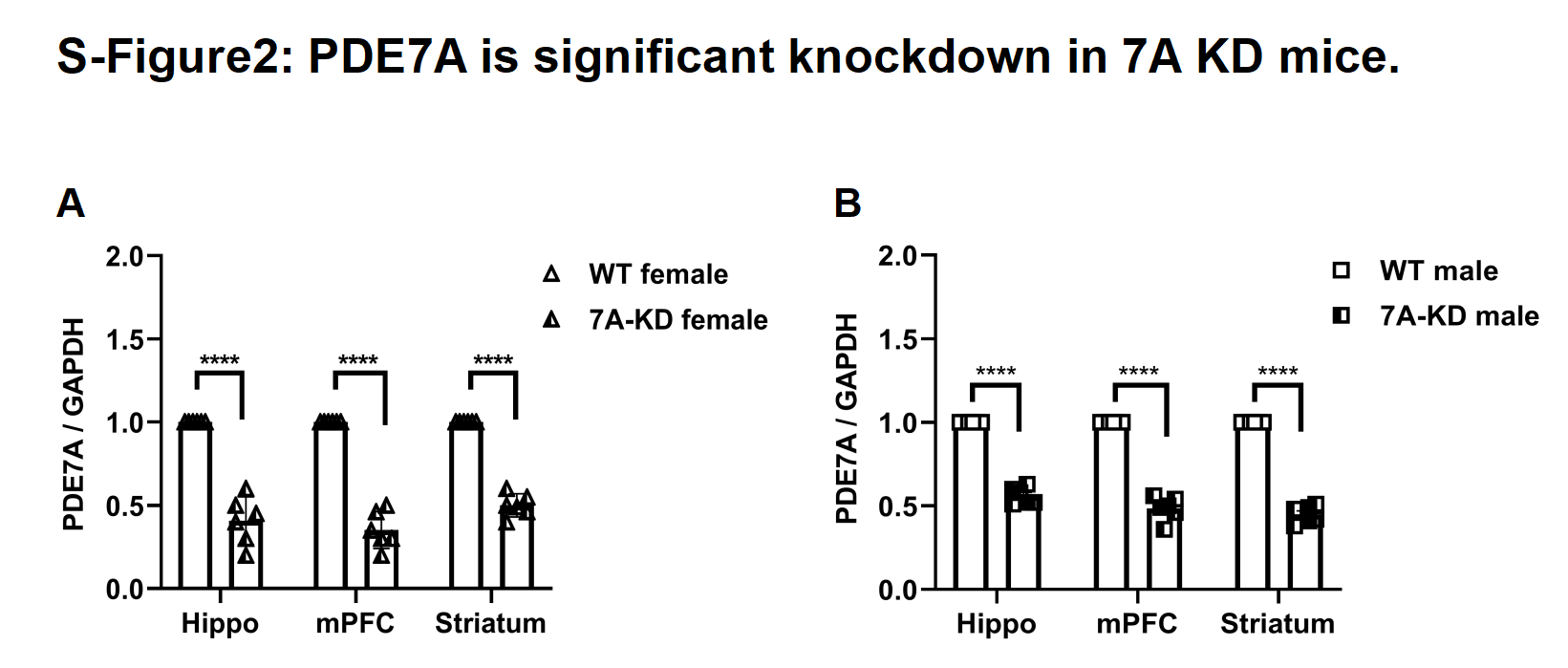


Knockdown efficiency in three brain regions (Hippo, mPFC and striatum) of female (A) and male (B) mice (t-test, Female-WT v.s. Female-KD, ****p < 0.0001 ; Male-WT v.s. Male-KD, ****p＜0.0001). n = 6 mice for each group. All data shown are means ± SEM, the ratio of PDE7A to the respective GAPDH in each lane was calculated for statistical analysis.

**Sup Fig3. BRL50481 have no influence on ethanol induced responsiveness and ethanol consumption in males.**

**
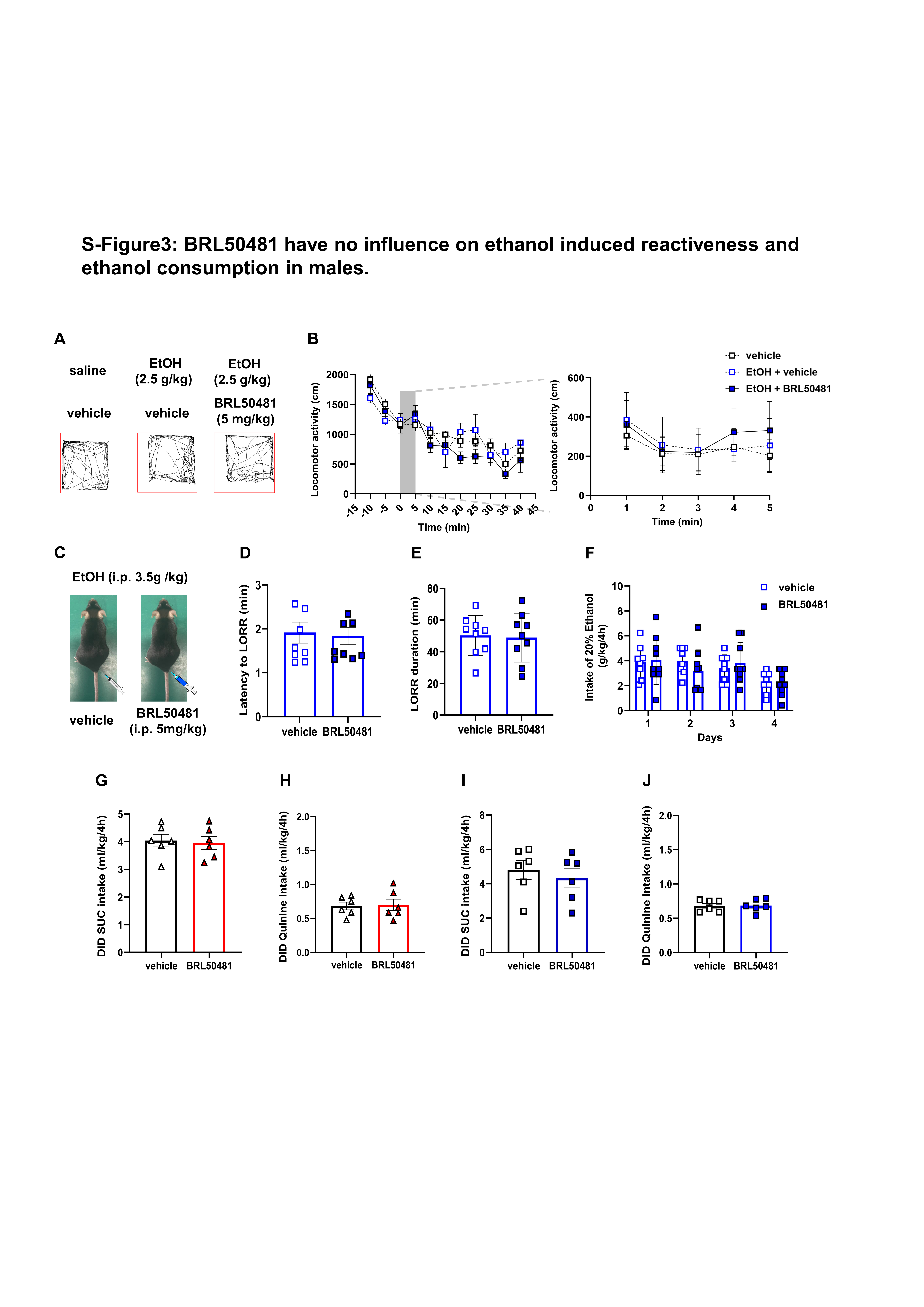
**

A) The locomotor trajectories of male mice were recorded at 5 minutes after intraperitoneal injection of saline or ethanol or ethanol (i.p. 2.5g/kg) with BRL50481 (i.p. 5mg/kg). B) Left, locomotor activity of C57BL/6J male mice for 15 min before injecting of saline or ethanol and 40 min following, shaded box highlights the locomotor activity is required analysis by minutes (One-way ANOVA, p > 0.05,). Right, locomotor activity of male mice analyzed in minute (One-way ANOVA, p > 0.05) C) BRL50481 was administered at a dose of 5 mg/kg, 2 hours prior to ethanol injection (i.p., 3.5 g/kg). D) Time required for the loss of righting reflex following administration of ethanol or ethanol combined with BRL50481 in male mice (t-test, p > 0.05). E) Time required for recovery of righting reflex following administration of ethanol or ethanol combined with BRL50481 in male mice (t-test, p > 0.05). F) Consumption of 20% ethanol in male mice of DID experiment (t-test, p > 0.05). G-H) The intake for 1% sucrose solution and 0.1mM quinine solution in female mice after administration of BRL50481 (t test, p > 0.05). I-J) The intake for 1% sucrose solution and 0.1mM quinine solution in male mice after administration of BRL50481 (t test, p > 0.05). n=8 mice for each group. All data shown are means ± SEM.

**Sup Fig4. PDE7 associated with cAMP-PKA /Epac2 pathway in other brain regions.**

**
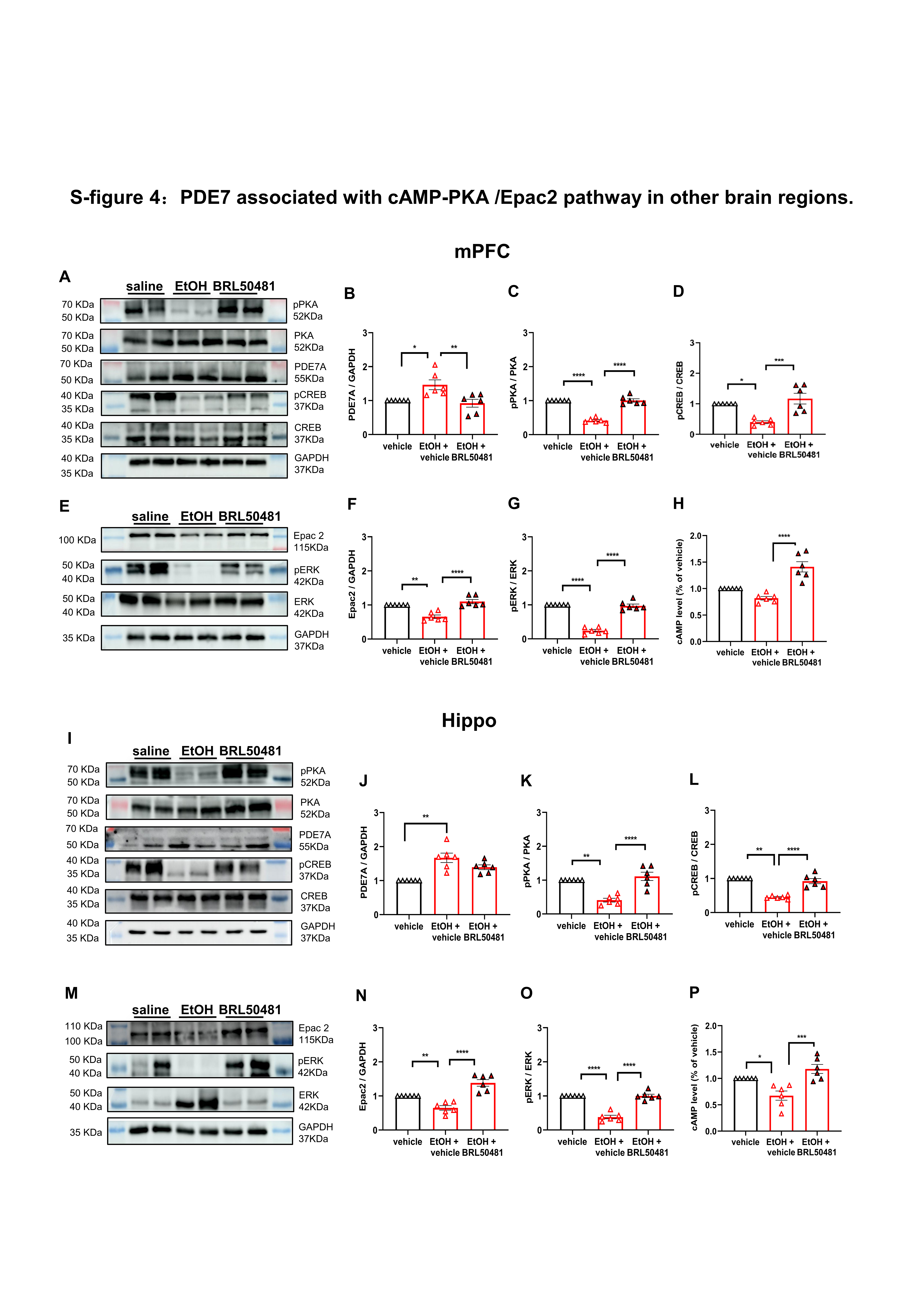
**

A-G) The protein levels of PDE7A, pPKA, PKA, pCREB, CREB, Epac2, pERK, and ERK in mPFC were measured after EtOH exposure and treatment with BRL50481 (One-way ANOVA, PDE7A, F(2, 12) = 2.78; pPKA/PKA, F(2, 12) = 9.23; pCREB/CREB, F(2, 12) = 12.75; Epac2, F(2, 12) = 2.78; pERK/ERK, F(2, 12) = 2.94; *p < 0.05, **p < 0.01, ****P＜0.0001). H) Elisa was used to measure cAMP level in the mPFC. (One-way ANOVA, F(2, 12) = 9.46, ****P＜0.0001). I-O) The protein levels of PDE7A, pPKA, PKA, pCREB, CREB, Epac2, pERK, and ERK in hippocampus were measured after EtOH exposure and treatment with BRL50481 (One-way ANOVA, PDE7A, F(2, 12) = 3.46; pPKA/PKA, F(2, 12) = 4.16; pCREB/CREB, F(2, 12) = 5.13; Epac2, F(2, 12) = 11.31; pERK/ERK, F(2, 12) = 2.70; *p < 0.05, **p < 0.01, ***P＜0.001, ****P＜0.0001). P) Elisa was used to measure cAMP level in the hippocampus. (One-way ANOVA, F(2, 12) = 2.98, *p < 0.05, ***P＜0.001,). n=6 mice for each group. All data shown are means ± SEM, the ratio of PDE7A to the respective GAPDH in each lane was calculated for statistical analysis.
